# Targeting innate immunity to modulate bone metabolism: a novel strategy for osteoporosis treatment

**DOI:** 10.3389/fragi.2026.1750450

**Published:** 2026-03-20

**Authors:** Wenjie Kou, Xiaomin Lu, Zhe Zhang, Kaiwen Liu, Zhihuan Liu, Bin Jiang, Hongping Wang, Jishu Li, Hu Lu, Chenglong Guo, Linzhong Cao, Xiaogang Zhang

**Affiliations:** 1 Gansu University of Traditional Chinese Medicine, Lanzhou, Gansu, China; 2 Longnan Maternal and Child Health Hospital, Longnan, Gansu, China; 3 Affiliated Hospital of Gansu University of Traditional Chinese Medicine, Lanzhou, Gansu, China

**Keywords:** bone metabolism, complement system, innate immunity, macrophages, osteoporosis, targeted therapy, trained immunity

## Abstract

Osteoporosis is a systemic metabolic bone disorder characterized by reduced bone mass and impaired microarchitecture, with its core pathological mechanism being an imbalance between bone formation and resorption. Traditional therapies targeting osteoblast/osteoclast function have limited efficacy and safety concerns. Recent osteoimmunology advances reveal that the innate immune system regulates bone homeostasis via intercellular interactions, cytokine networks, and metabolic reprogramming. This systematic review examines the roles of innate immune cells (macrophages, neutrophils, NK cells, DCs), complement system, and emerging pathways (trained immunity, mitochondrial symbiosis disruption) in osteoporosis. It summarizes therapeutic strategies (immunometabolic modulators, complement antagonists, cytokine-targeted drugs, TCM components) and outlines challenges (target specificity, clinical translation) and future directions, providing theoretical foundations for novel OP treatments.

## Introduction

Osteoporosis (OP) is the most prevalent metabolic bone disorder globally. Data from the International Osteoporosis Foundation indicate approximately 200 million people worldwide are affected, with postmenopausal women and elderly men constituting high-risk populations (Luo et al., 2025). Its core characteristics include reduced bone mineral density (BMD) and deteriorated trabecular microarchitecture, leading to increased bone fragility and susceptibility to vertebral and hip fractures. This not only diminishes patients’ quality of life but also exacerbates the societal and medical burden. Population aging further elevates the prevention and treatment of this disease as a critical public health challenge (Manoj et al., 2023). Bone metabolism relies on the dynamic equilibrium between osteoblast-mediated bone formation and osteoclast-mediated bone resorption, jointly regulated by endocrine hormones, local cytokines, and immune cells. Traditional treatments fall into two categories: inhibiting resorption (e.g., bisphosphonates, denosumab) and promoting formation (e.g., teriparatide). However, limitations exist: long-term bisphosphonate use may cause osteonecrosis of the jaw and atypical femoral fractures; denosumab leads to rebound bone loss upon discontinuation; teriparatide requires daily injections and carries potential carcinogenic risks. Thus, safer therapeutic strategies are urgently needed (Reid and Billington, 2022). Bone immunology reveals that the immune and skeletal systems are integrated through an “immune-bone” network. This network is centered on innate immunity, where diverse components—including immune cells (macrophages, neutrophils, NK cells, DCs), complement system, and emerging regulatory pathways (trained immunity, mitochondrial symbiosis)—interact synergistically: immune cells secrete cytokines to modulate osteoblast/osteoclast activity, complement activation products (C3a/C5a) disrupt bone cell function, and metabolic reprogramming (e.g., glycolysis, mitochondrial dysfunction) reshapes immune cell phenotypes, collectively driving bone metabolic balance or imbalance. Innate immunity (involving cells like macrophages and neutrophils, along with molecules such as complement and cytokines) maintains bone homeostasis during physiological states, while pathological states (e.g., estrogen deficiency, aging) disrupt this equilibrium (e.g., estrogen deficiency induces macrophage secretion of pro-inflammatory factors to activate osteoclasts). This network serves as a pivotal hub for inflammation and bone metabolic imbalance, making innate immunity a novel therapeutic target for OP treatment (Guo Y. et al., 2025). This article will explore three dimensions: the mechanisms of innate immunity, emerging regulatory pathways, and targeted therapeutic strategies.

## Mechanisms of innate immune cells in osteoporosis

Innate immune cells constitute a vital component of the bone microenvironment, regulating the differentiation, function, and survival of osteoblasts and osteoclasts through direct contact or secretion of effector molecules (Xu J. et al., 2023). Under the pathological conditions of osteoporosis, the phenotype, function, and metabolic characteristics of innate immune cells undergo abnormal alterations, becoming core drivers of bone metabolic imbalance. This section will focus on elucidating the roles and mechanisms of macrophages, neutrophils, natural killer cells, and dendritic cells in osteoporosis (Ding et al., 2025).

## Macrophages: dual regulators of bone metabolism

Macrophages constitute the most abundant innate immune cells in the bone microenvironment. Functionally categorized as classical activated (M1) or alternative activated (M2) macrophages, these two subtypes exert diametrically opposed effects on bone metabolism (Yunna et al., 2020). Under physiological conditions, M2 macrophages promote mesenchymal stem cell (MSC) differentiation into osteoblasts and suppress osteoclastogenesis by secreting factors such as transforming growth factor-β (TGF-β) and bone morphogenetic protein-2 (BMP-2) (Wei et al., 2023). Conversely, M1 macrophages are activated under stress conditions such as infection or trauma. They secrete pro-inflammatory factors like TNF-α, IL-6, and IL-1β, which activate the nuclear factor-κB (NF-κB) signaling pathway and promote osteoclastogenesis (Li et al., 2023).

In osteoporosis, the polarization balance of macrophages is disrupted, with M1 polarization becoming dominant, making it a key driver of bone loss (Yang et al., 2024). Taking postmenopausal osteoporosis as an example, estrogen deficiency induces M1 polarization of macrophages by activating the Toll-like receptor 4 (TLR4)/Myeloid Differentiation Factor 88 (MyD88) signaling pathway. TNF-α secreted by M1 macrophages directly binds to TNF receptors on osteoclast precursor cells, activating downstream c-Jun N-terminal kinase (JNK) signaling to promote expression of the key osteoclast differentiation transcription factor NFATc1 (Cytoplasmic1). Concurrently, TNF-α further enhances osteoclastogenesis by upregulating RANKL expression on osteoblast surfaces (Li et al., 2019). Furthermore, IL-6 secreted by M1 macrophages promotes osteoclast differentiation by activating the signal transducer and activator of transcription 3 (STAT3) pathway in synergy with RANKL, while simultaneously suppressing osteoblast function (Chen et al., 2022a).

Beyond disrupted polarization balance, abnormal efferocytosis in macrophages also contributes to osteoporosis pathogenesis. Efferocytosis is a critical process by which macrophages clear apoptotic cells (e.g., apoptotic osteoblasts and osteoclasts), maintaining bone microenvironment homeostasis by releasing anti-inflammatory factors such as TGF-β and IL-10 (Batoon et al., 2024). Studies reveal significantly impaired efferocytic function in bone macrophages of aged mice, leading to accumulation of apoptotic osteoblasts and triggering chronic inflammatory responses (Sinder et al., 2017). Further studies indicate that age-related downregulation of SIRT6 induces expression of the “anti-phagocytic signal molecule” CD47 on apoptotic osteoblast surfaces. By binding to macrophage surface signal regulatory protein α (SIRPα), CD47 inhibits phagocytosis, ultimately reducing bone formation (Xu R. et al., 2023). Moreover, in diabetes-associated osteoporosis, a hyperglycemic environment exacerbates bone loss by activating the ROS/MAPK/NF-κB/NLRP3 inflammasome pathway, thereby suppressing macrophage phagocytic function (An et al., 2019).

In recent years, the discovery of bone-specific macrophages has provided new targets for osteoporosis research. Osteomacs are resident macrophages located on the bone surface, constituting one-sixth of total bone marrow cells. They regulate bone formation by enveloping osteoblasts through the formation of a cellular “canopy” structure (Chang et al., 2008). Studies indicate that Osteomacs promote osteoblast proliferation by secreting platelet-derived growth factor (PDGF). Concurrently, they maintain bone remodeling equilibrium by phagocytosing bone resorption products generated by osteoclasts (Weivoda and Bradley, 2023). In an ovariectomy (OVX)-induced osteoporosis mouse model, Osteomacs numbers significantly decreased, and their PDGF secretion capacity declined, leading to osteoblast functional suppression. Supplementing with recombinant PDGF partially restored bone mass, suggesting Osteomacs may represent a potential therapeutic target for osteoporosis (Batoon et al., 2021).

## Neutrophils: the “accelerator” of bone resorption

Neutrophils are the most abundant innate immune cells in peripheral blood, traditionally recognized primarily for their role in combating bacterial infections (Liew and Kubes, 2019). However, recent studies reveal that neutrophils also play a crucial role in maintaining bone homeostasis. Particularly in osteoporosis, neutrophils act as key “accelerators” of bone resorption by releasing NETs, pro-inflammatory factors, and proteases (Schneider et al., 2024).

NETs are reticular structures composed of DNA, histones, and granular proteins (e.g., neutrophil elastase, matrix metalloproteinase-9) released by activated neutrophils, initially discovered for trapping pathogens (Qiu et al., 2025). Studies reveal significantly elevated NET levels in peripheral blood and bone marrow of ovariectomized (OVX) mouse models, with NETs directly promoting osteoclast generation (Guo Y. et al., 2025). Mechanistically, neutrophil elastase within NETs degrades osteoprotegerin (OPG) in the bone microenvironment, disrupting the RANKL/OPG balance and enhancing osteoclastogenesis. Concurrently, histones in NETs activate the TLR2/4 signaling pathway on osteoclast precursor surfaces, inducing NFATc1 expression and further promoting osteoclast differentiation. Furthermore, NETs recruit additional inflammatory cells to the bone microenvironment by releasing factors such as IL-1β and IL-8, establishing a vicious cycle of “inflammation-bone resorption” (Schneider et al., 2024).

Beyond NETs, other effector molecules secreted by neutrophils also contribute to osteoporosis pathogenesis. For instance, Matrix Metalloproteinase-8 (MMP-8) released by neutrophils degrades collagen in the bone matrix, promoting bone resorption. Neutrophil-derived Reactive Oxygen Species (ROS) can damage osteoblast DNA through oxidative stress, inhibiting osteoblast proliferation and differentiation (Chang and Nguyen, 2021). In senile osteoporosis, neutrophil ROS production capacity is significantly enhanced. The released ROS can induce osteocyte senescence by activating the p53 signaling pathway in osteocytes, leading to reduced bone formation (Iantomasi et al., 2023).

Notably, the role of neutrophils in bone metabolism is time-dependent. During the early stages of bone injury repair, transient neutrophil infiltration promotes bone healing by releasing chemokines (e.g., CXCL1, CXCL2) that recruit MSCs and osteoblast precursors. Conversely, in chronic inflammatory states (such as osteoporosis), sustained neutrophil activation exacerbates bone loss (Cai et al., 2025). Therefore, therapeutic strategies targeting neutrophils require precise regulation of their activation timing and intensity to avoid disrupting normal bone repair processes.

## Natural killer cells: the “Guardians” of bone homeostasis

Natural killer (NK) cells are key players in innate immunity responsible for eliminating virus-infected cells and tumor cells. They exert their functions by releasing perforin, granzyme, and cytokines (Thompson and Cao, 2024). Recent studies reveal that NK cells also participate in bone metabolism regulation, exhibiting a “dual role” in osteoporosis: on one hand, they inhibit bone formation by directly killing osteoblast precursors; on the other hand, the interferon-γ (IFN-γ) secreted by NK cells suppresses osteoclast generation. The disruption of this dual-action equilibrium constitutes a significant mechanism in osteoporosis pathogenesis (Kaur et al., 2023).

In postmenopausal osteoporosis, estrogen deficiency enhances NK cell cytotoxicity by downregulating inhibitory receptors (e.g., KIR2DL1) on their surface (Qi et al., 2025). Activated NK cells recognize stress-associated ligands (e.g., MICA/B) on osteoblast precursor surfaces, releasing perforin and granzyme B to induce osteoblast precursor apoptosis, thereby reducing bone formation (Meng et al., 2025). Additionally, estrogen deficiency promotes NK cell migration to bone marrow, where they secrete factors like TNF-α and IL-6 to synergistically enhance osteoclastogenesis with macrophages (Sun et al., 2023). Clinical studies reveal significantly elevated NK cell proportions and cytotoxicity in peripheral blood of postmenopausal osteoporosis patients, negatively correlated with BMD, suggesting NK cells may serve as potential diagnostic and therapeutic targets for osteoporosis (Jones et al., 2023).

However, under certain pathological conditions, NK cells may also exert bone-protective effects. For instance, in inflammatory bowel disease-associated osteoporosis, IFN-γ secreted by NK cells inhibits osteoclast formation by suppressing the STAT1 signaling pathway in osteoclast precursors and downregulating NFATc1 expression (Kaur et al., 2023). This dual role may be related to NK cell tissue origin, activation status, and microenvironmental signals. For instance, spleen-derived NK cells (SpNK) primarily promote bone repair via Ncr1 (Natural Cytotoxicity Receptor 1) signaling, whereas thymus-derived NK cells (ThNK) inhibit bone regeneration through Klrk1 (Killer Cell Lectin-Like Receptor K1) and perforin (Dastagir et al., 2022). Therefore, elucidating the roles and mechanisms of distinct NK cell subsets in osteoporosis is crucial for developing precision-targeted therapeutic strategies.

## Dendritic cells: the “Bridge” of bone immunity

Dendritic cells (DCs) serve as pivotal cells linking innate and adaptive immunity. They initiate adaptive immune responses by capturing, processing, and presenting antigens to T cells (Balan et al., 2019). Recent studies indicate that DCs also play a vital role in regulating bone metabolism, particularly in autoimmune-related osteoporosis (such as rheumatoid arthritis-associated osteoporosis). Here, DCs indirectly influence bone metabolism by activating T cell immune responses.

In RA-related osteoporosis, DCs activate Th17 cells by presenting self-antigens (e.g., type II collagen) from the synovium (Umbreen et al., 2022). IL-17 secreted by Th17 cells promotes osteoclastogenesis by upregulating RANKL expression on the surface of osteoblasts and synovial fibroblasts. Concurrently, IL-17 inhibits osteoblast differentiation by activating the NF-κB signaling pathway (Cavalla et al., 2021). Furthermore, IL-23 secreted by DCs enhances IL-17-mediated bone resorption by sustaining Th17 cell survival and proliferation (Puig et al., 2022). Clinical studies reveal significantly elevated DC proportions and IL-23 secretion levels in peripheral blood of rheumatoid arthritis patients, positively correlated with bone erosion severity.

Beyond indirect regulation, DCs directly influence osteoclastogenesis (Li L. et al., 2023). *In vitro* studies indicate that DCs promote osteoclast differentiation by expressing RANKL, which directly binds to RANK on osteoclast precursor cells. Furthermore, IL-1β secreted by DCs enhances osteoclastogenesis by activating the NF-κB signaling pathway in osteoclast precursors, synergistically with RANKL (Hu et al., 2022; Zhang W. et al., 2025). In postmenopausal osteoporosis, estrogen deficiency exacerbates bone loss by activating the TLR9 signaling pathway on DCs, inducing secretion of factors such as IL-1β and TNF-α (Cline-Smith et al., 2020).

**FIGURE 1:**
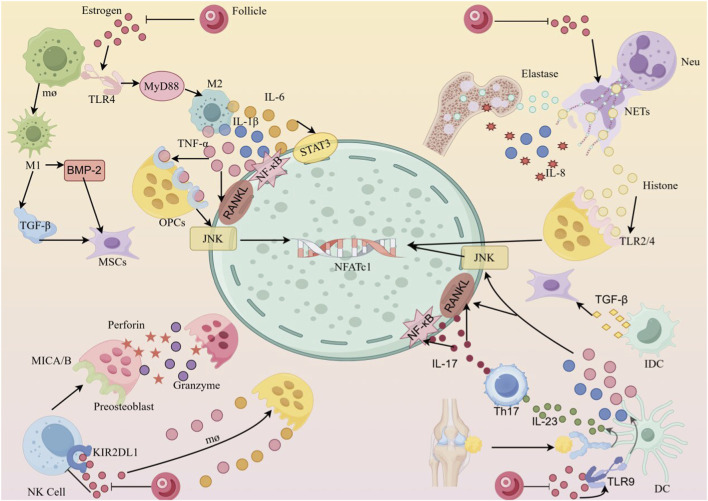
The mechanism of innate immune cells in OP Key pathways through which four types of core innate immune cells (macrophages, neutrophils, NK cells, DCs) regulate bone metabolism. These cells form a synergistic regulatory network through a “direct-action cytokine network”: ① Macrophages (M1/M2 subtypes): M1 macrophages secrete TNF-α and IL-6 via the TLR4/MyD88 pathway, activating NF-κB/STAT3 signaling to promote osteoclast (OC) differentiation; M2 macrophages secrete TGF-β and BMP-2, inducing mesenchymal stem cells (MSCs) to differentiate into osteoblasts. ② Neutrophils (Neu): release NETs containing elastase and histones, which degrade OPG and activate TLR2/4 pathways, disrupting the RANKL/OPG balance. ③ NK cells: downregulate via KIR2DL1 to enhance cytotoxicity, release perforin/granzyme to kill osteoblast precursors, and secrete TNF-α to synergistically promote osteoclast activity. ④ DCs: activated through TLR9, secrete IL-1β and IL-23, inducing Th17 cells to produce IL-17, which further upregulates RANKL expression in osteoblasts.

Notably, the maturation state of DCs significantly influences bone metabolism. Immature DCs promote MSC differentiation toward osteoblasts by secreting TGF-β, whereas mature DCs primarily suppress bone formation by activating adaptive immune responses (Favaro et al., 2016; Shahir et al., 2020). Thus, regulating DC maturation may represent a novel therapeutic strategy for osteoporosis. For instance, studies have demonstrated that TLR9 antagonists inhibit DC maturation, reduce IL-23 secretion, suppress Th17 cell activation, and alleviate bone loss in rheumatoid arthritis-associated osteoporosis mouse models (Fischer et al., 2018) (Figure 1).

## Mechanisms of innate immunity in regulating bone metabolism: shared pathways

Across the aforementioned innate immune components (immune cells, complement system), several core pathways are repeatedly involved in bone metabolism regulation, forming a shared regulatory network: 1) NF-κB signaling pathway: activated by pro-inflammatory cytokines (TNF-α, IL-6) or complement products (C5a), it promotes osteoclast differentiation via upregulating NFATc1; 2) RANKL/OPG balance: most innate immune mediators (e.g., M1 macrophages, NETs, C5a) disrupt this balance by upregulating RANKL or downregulating OPG, enhancing bone resorption; 3) metabolic reprogramming: glycolysis activation in M1 macrophages/neutrophils and mitochondrial dysfunction in bone cells are common pathological features, linking immune activation to bone loss. These shared pathways provide potential pan-targets for therapeutic development, while cell-specific mechanisms (e.g., macrophage efferocytosis, NK cell dual roles) offer precision-targeting opportunities.

## Mechanisms of the complement system in osteoporosis

The complement system is a crucial component of innate immunity, comprising over 30 soluble proteins and membrane-bound receptors. Its primary functions include eliminating pathogens and apoptotic cells while maintaining immune homeostasis (DeVaughn et al., 2024). Traditionally, the complement system was considered to function primarily in the humoral environment. However, recent studies indicate that it also plays a crucial role in regulating bone metabolism, particularly in osteoporosis. Complement activation products (such as C3a and C5a) bind to complement receptors on the surface of bone cells, directly or indirectly modulating bone formation and resorption, thereby becoming key drivers of bone metabolic imbalance (Tu et al., 2010; Li and Cui, 2025).

## Complement system activation and bone metabolic regulation

The complement system can be activated through the classical pathway, lectin pathway, and alternative pathway, ultimately forming the membrane attack complex (MAC) to mediate cell lysis (Ellsworth et al., 2024). Within the bone microenvironment, activated complement system products (e.g., C3a, C5a) regulate bone metabolism by binding to complement receptors (e.g., C3aR, C5aR1) on the surfaces of osteoblasts, osteoclasts, and immune cells. Under physiological conditions, moderate activation of the complement system maintains bone homeostasis (Modinger et al., 2018b). For instance, C3a activates the downstream ERK1/2 signaling pathway by binding to C3aR on osteoblast surfaces, thereby promoting osteoblast proliferation and differentiation (Ming et al., 2025); conversely, C5a reduces osteoclastogenesis by suppressing NFATc1 expression in osteoclast precursors (Pimenta-Lopes et al., 2023). Furthermore, the complement system maintains bone homeostasis by clearing apoptotic cells from the bone microenvironment, thereby preventing chronic inflammatory responses.

In osteoporosis, excessive complement activation exacerbates bone loss through multiple mechanisms. Taking postmenopausal osteoporosis as an example, estrogen deficiency upregulates complement C3 expression in bone marrow, activating the alternative pathway and elevating C3a and C5a levels. C5a binds to C5aR1 on osteoblast surfaces, activating the downstream JNK signaling pathway to induce osteoblast apoptosis. Concurrently, C5a promotes osteoclastogenesis by upregulating RANKL expression on osteoblast surfaces (Kosa et al., 2009; Modinger et al., 2018a). Clinical studies have revealed significantly elevated serum C3 and C5a levels in PMOP mice, negatively correlated with BMD, suggesting that complement system activation may serve as a diagnostic biomarker for osteoporosis (Guha et al., 2025; MacKay et al., 2018).

## C5a/C5aR1 axis: a key target for osteoporosis treatment

Among complement system activation products, C5a and its receptor C5aR1 exhibit the most well-defined roles in osteoporosis, making them a research hotspot in this field. C5aR1 is a G protein-coupled receptor widely expressed on the surface of osteoblasts, osteoclasts, macrophages, and other cells. By binding to C5a, it activates downstream signaling pathways to regulate cellular functions (Ruocco et al., 2022).

In osteoblasts, excessive activation of the C5a/C5aR1 axis inhibits osteoblast differentiation and induces apoptosis. Mechanistic studies indicate that C5a binding to C5aR1 on osteoblasts activates Gαi protein, thereby inhibiting adenylate cyclase activity and reducing intracellular cAMP levels. This suppresses expression of Runx2 (Runt-Related Transcription Factor 2), a key transcription factor for osteoblast differentiation. Simultaneously, C5a induces osteoblast apoptosis by activating the phospholipase C (PLC)/calcium/calmodulin-dependent protein kinase II (CaMKII) signaling pathway (Bulow et al., 2022). In an ovariectomized (OVX) mouse model, administration of a C5aR1 antagonist significantly suppressed osteoblast apoptosis, promoted bone formation, and restored bone mass.

Within osteoclasts, the C5a/C5aR1 axis promotes osteoclastogenesis through both direct and indirect mechanisms (Bulow et al., 2022). Regarding direct mechanisms, C5a binds to C5aR1 on osteoclast precursor surfaces, activating the NF-κB signaling pathway and promoting NFATc1 expression, thereby enhancing osteoclast differentiation. Indirectly, C5a activates macrophages to secrete proinflammatory factors such as TNF-α and IL-6, further boosting osteoclastogenesis (Serfecz et al., 2021). Studies revealed that C5aR1 knockout mice exhibited significantly reduced bone loss and decreased osteoclast numbers following ovariectomy (OVX), suggesting C5aR1 as a potential therapeutic target for osteoporosis (Kovtun et al., 2017).

Recent years have witnessed significant progress in drug development targeting the C5a/C5aR1 axis. For instance, the C5aR1 antagonist PMX53 significantly suppressed bone loss and increased BMD in an OVX mouse model. Furthermore, the novel oral C5aR1 antagonist avacopan has entered clinical trials in rheumatoid arthritis patients, demonstrating significant reductions in serum TNF-α and IL-6 levels alongside protective effects on bone metabolism (Pimenta-Lopes et al., 2023). These studies suggest that targeting the C5a/C5aR1 axis may represent a novel therapeutic strategy for osteoporosis.

## Interactions between the complement system and other signaling pathways

The complement system does not regulate bone metabolism independently but interacts extensively with other signaling pathways (such as RANKL/RANK/OPG and Wnt/β-catenin) to jointly maintain bone homeostasis. In osteoporosis, dysregulation of these complement-pathway interactions further exacerbates bone metabolic disorders.

The interaction between the complement system and the RANKL/RANK/OPG pathway is pivotal in regulating bone resorption. The RANKL/RANK/OPG pathway serves as the core regulatory pathway for osteoclastogenesis. RANKL secreted by osteoblasts binds to RANK on the surface of osteoclast precursors, promoting osteoclast differentiation. Conversely, OPG, acting as a decoy receptor for RANKL, inhibits osteoclast formation by binding to RANKL (And Alternative Medicine, 2020). Studies reveal that C5a disrupts the RANKL/OPG balance by upregulating RANKL expression on osteoblast surfaces while downregulating OPG expression, thereby promoting osteoclastogenesis (Bergdolt et al., 2017). Mechanistically, C5a induces RANKL gene transcription by activating the NF-κB signaling pathway in osteoblasts. Concurrently, C5a suppresses OPG expression by inhibiting the Wnt/β-catenin signaling pathway, further enhancing osteoclastogenesis.

The interaction between the complement system and the Wnt/β-catenin pathway primarily influences osteogenesis. The Wnt/β-catenin pathway is a key regulator of osteoblast differentiation. Upon binding to its cell surface receptor, Wnt ligands inhibit β-catenin degradation, promote its nuclear translocation, and activate the expression of osteoblast differentiation-related genes. Research indicates that C5a promotes β-catenin degradation by activating glycogen synthase kinase-3β (GSK-3β), thereby inhibiting osteoblast differentiation (Yang et al., 2025). In an ovariectomized (OVX) mouse model, administration of a C5aR1 antagonist significantly activated the Wnt/β-catenin pathway, promoted osteoblast differentiation, and restored bone mass. Conversely, a Wnt/β-catenin pathway inhibitor reversed the bone-protective effects of the C5aR1 antagonist, suggesting the complement system contributes to osteoporosis pathogenesis by inhibiting the Wnt/β-catenin pathway.

**FIGURE 2:**
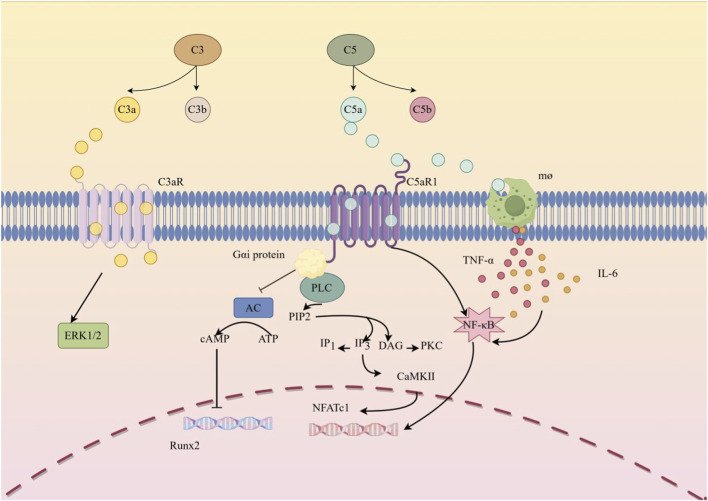
Mechanisms by which the complement system acts in OP Dual regulation of bone metabolism and signal interactions following complement system activation: ① Complement activation products (C3a, C5a): C3a binds to osteoblast C3aR, activating the ERK1/2 pathway to promote osteogenesis; C5a binds to C5aR1, inhibiting adenylate cyclase (AC) via Gαi protein, reducing cAMP levels and suppressing Runx2 expression, while simultaneously activating the PLC/CaMKII pathway to induce osteoblast apoptosis. ② Inflammatory amplification effect: C5a activates macrophages (mo) to secrete TNF-α and IL-6, which enhance osteoclast formation through paracrine action. ③ Core signaling nodes: The figure clearly labels second messengers such as PIP2, IP3/DAG, and PKC, linking complement receptors with downstream transcription factors like NFATc1 and Runx2, visually illustrating the interaction mechanism between the complement system and osteocyte signaling pathways, explaining the pathological logic of ‘excessive complement activation → inhibited osteogenesis and enhanced osteoclast activity.”

Furthermore, the complement system interacts with inflammasome signaling pathways (MacKay et al., 2018). The NLRP3 (NOD-Like Receptor Pyrin Domain-Containing Protein 3) inflammasome, a multiprotein complex, promotes IL-1β maturation and secretion upon activation. Research indicates that C5a enhances osteoclastogenesis by activating NLRP3 inflammasomes in osteoclast precursors, thereby promoting IL-1β secretion. NLRP3 knockout mice exhibited significantly reduced bone loss following ovariectomy (OVX) and demonstrated insensitivity to C5a-induced osteoclastogenesis, suggesting the complement system contributes to osteoporosis pathogenesis by activating NLRP3 inflammasomes (Haggadone et al., 2016; Li et al., 2025) (Figure 2).

## Emerging mechanisms of innate immunity in regulating bone metabolism

With the advancement of bone immunology research, beyond traditional cytokine-mediated mechanisms, novel regulatory pathways such as trained immunity and interruption of mitochondrial symbiosis have been progressively revealed. These discoveries offer fresh perspectives for understanding the pathogenesis of osteoporosis and developing novel therapeutic strategies (Vuscan et al., 2024). This section will focus on elucidating the roles of these emerging mechanisms in innate immunity regulation of bone metabolism.

## Trained immunity: innate immunity's “memory” and bone metabolic regulation

Trained immunity refers to the enhanced response capacity acquired by innate immune cells (e.g., macrophages, monocytes) following initial exposure to pathogens or inflammatory stimuli. This capacity arises through epigenetic and metabolic reprogramming, with the resulting “memory” effect persisting for weeks to months (Dominguez-Andres et al., 2023). Traditionally viewed as primarily involved in anti-infective immunity, recent studies reveal that trained immunity also plays a crucial role in bone metabolism regulation. Particularly in chronic inflammation-associated osteoporosis (e.g., senile osteoporosis, diabetes-related osteoporosis), dysregulation of innate immune cell function mediated by trained immunity emerges as a key driver of bone metabolic imbalance (Rahmani et al., 2024).

In senile osteoporosis, bone marrow monocytes (BMMs) exhibit an immunologically trained phenotype, with significantly enhanced responsiveness to subsequent inflammatory stimuli (Benjaskulluecha et al., 2024). Studies reveal that in aged mice, histone H3K4me3 (an activation-type epigenetic mark) accumulates in the promoter regions of proinflammatory factor genes like IL-6 and TNF-α in BMMs, elevating their basal expression levels. Concurrently, enhanced glycolytic metabolism and increased lactate production in aged BMMs further promote proinflammatory factor secretion (Chen et al., 2023). Regarding the duration of this memory in the bone marrow niche, current evidence suggests that the epigenetic reprogramming of BMMs can persist for 4–12 weeks in preclinical models (Dominguez-Andres et al., 2023; Rahmani et al., 2024). This long-lasting epigenetic modification implies that short-term early interventions (e.g., targeted inhibition of DNMT1 or ROS scavenging) could potentially reverse or normalize the trained immune phenotype of BMMs, thereby exerting long-term benefits for bone health by interrupting the “inflammation-bone resorption” vicious cycle. This reprogramming persistence also highlights the potential of timed, early therapeutic interventions to modulate innate immune memory and prevent chronic bone loss in high-risk populations (e.g., premenopausal women at risk of PMOP or elderly individuals). This trained immune phenotype perpetuates osteoclastogenesis through an “inflammation-bone resorption” vicious cycle, accelerating bone loss. Mechanistically, age-related increased ROS production activates DNA methyltransferase 1 (DNMT1), inducing epigenetic reprogramming in BMMs to form the trained immune phenotype. Conversely, treatment with ROS scavengers or DNMT1 inhibitors reverses BMMs’ trained immune phenotype, reduces proinflammatory cytokine secretion, and inhibits osteoclastogenesis (Chen et al., 2024).

In diabetes-associated osteoporosis, a high-sugar environment induces BMMs to develop an immune training phenotype, accelerating bone loss. Studies reveal that high-glucose stimulation activates the TLR4/MyD88 signaling pathway, inducing metabolic reprogramming in BMMs characterized by enhanced glycolysis and reduced mitochondrial oxidative phosphorylation. Simultaneously, high glucose promotes histone acetylation of proinflammatory factor genes by upregulating histone acetyltransferase p300, thereby enhancing their expression. This trained immune phenotype enables BMMs to secrete increased TNF-α and IL-6 upon subsequent inflammatory stimulation, thereby promoting osteoclastogenesis (Cao et al., 2021). Clinical studies reveal significantly elevated glycolysis levels in peripheral blood mononuclear cells (PBMCs) from diabetic patients, negatively correlated with bone mineral density (BMD). This suggests trained immunity may serve as a diagnostic and therapeutic target for diabetes-related osteoporosis (Brandt et al., 2024).

**FIGURE 3:**
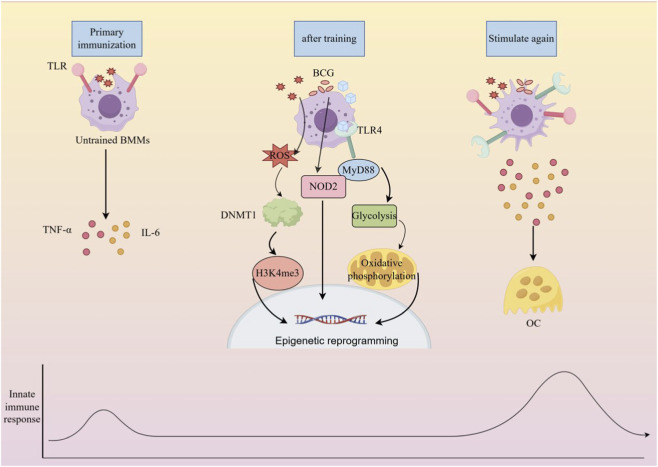
Trained immunity-mediated regulatory mechanisms in bone metabolism pathway of trained immunity-induced ‘epigenetic reprogramming - metabolic remodeling - bone metabolism imbalance’: ① Initial stimulation phase (left): Initial stimuli such as BCG or high glucose activate ROS/DNMT1 signaling in bone marrow monocytes (BMMs) through the TLR4/MyD88 or NOD2 pathways; ② Reprogramming phase (middle): Reprogramming occurs at the epigenetic level (H3K4me3 enrichment) and metabolic level (enhanced glycolysis, reduced oxidative phosphorylation), forming a ‘trained immunity phenotype’; ③ Effector phase (right): Upon re-stimulation, trained BMMs secrete large amounts of TNF-α and IL-6, continuously promoting osteoclast (OC) formation. The figure clearly distinguishes the metabolic differences between ‘untrained BMMs’ and ‘trained BMMs’, and highlights key regulatory molecules such as NOD2 and DNMT1, explaining the molecular basis of the memory effect of trained immunity and the core mechanism by which short-term initial stimulation leads to long-term bone loss.

Notably, trained immunity does not solely exert bone-destructive effects; under specific conditions, it can also exert bone-protective effects by enhancing osteoblast function. For instance, after training immunotherapy induction in mice using *Bacillus* Calmette-Guérin (BCG) or β-glucan, the BMP-2 secretion levels of bone marrow macrophages significantly increased, promoting MSC differentiation into osteoblasts and enhancing BMD (Xu et al., 2024). Mechanistically, BCG activates the NOD2 (Nucleotide-Binding Oligomerization Domain 2) signaling pathway, inducing epigenetic reprogramming in macrophages and upregulating BMP-2 gene expression (Kleinnijenhuis et al., 2012). These studies suggest that modulating the direction of trained immunity (pro-inflammatory vs. anti-inflammatory; pro-resorptive vs. pro-formative) may represent a novel therapeutic strategy for osteoporosis (Figure 3).

## Disruption of mitochondrial symbiosis: metabolic reprogramming and bone metabolic dysregulation

Mitochondria serve as cellular “powerhouses” while also functioning as pivotal regulators of innate immunity. They contribute to the activation and functional regulation of innate immune cells by generating reactive oxygen species (ROS), releasing mitochondrial DNA (mtDNA), and secreting metabolic byproducts such as succinate and citrate (Fang et al., 2016). Recent studies have introduced the concept of “disrupted mitochondrial symbiosis,” where the symbiotic relationship between mitochondria and host cells is broken, leading to mitochondrial dysfunction that subsequently impacts cellular metabolism and immune function. In osteoporosis, mitochondrial symbiosis disruption mediates metabolic reprogramming of innate immune cells and bone cell dysfunction, emerging as a key mechanism for bone metabolic imbalance.

In macrophages, mitochondrial symbiosis disruption promotes M1 polarization through metabolic reprogramming, exacerbating bone resorption (Zhang H. et al., 2025). Studies reveal that bone marrow macrophages from ovariectomized (OVX) mice exhibit significantly reduced mitochondrial membrane potential, increased mitochondrial fission, and heightened ROS production. Concurrently, these macrophages demonstrate weakened fatty acid oxidation metabolism and enhanced glycolysis, leading to increased proinflammatory factor secretion (Lai et al., 2025). Mechanistically, estrogen deficiency inhibits SIRT3 (a mitochondrial deacetylase) expression, elevating acetylation levels of mitochondrial respiratory chain complex I and suppressing its activity, thereby causing mitochondrial dysfunction. Conversely, SIRT3 overexpression restores macrophage mitochondrial function, suppresses M1 polarization, and mitigates OVX-induced bone loss (Li et al., 2021). Furthermore, disrupted mitochondrial symbiosis releases mtDNA, activating the cGAS-STING (Cyclic GMP-AMP Synthase-Stimulator of Interferon Genes) signaling pathway to promote macrophage IFN-β secretion, thereby exacerbating inflammation.

In osteoblasts, disrupted mitochondrial symbiosis inhibits differentiation and function by suppressing energy metabolism and inducing oxidative stress. Osteoblast differentiation and function demand substantial ATP, with mitochondrial oxidative phosphorylation serving as the primary ATP production pathway (Zhou et al., 2024). Studies reveal that aged mouse osteoblasts exhibit reduced mitochondrial numbers, decreased respiratory chain complex activity, and insufficient ATP production, leading to downregulation of key osteoblast differentiation genes (e.g., Runx2, Osterix). Concurrently, increased ROS production from disrupted mitochondrial symbiosis can oxidatively damage osteoblast DNA, inducing osteoblast senescence and further inhibiting bone formation (Siddiqi et al., 2014). Mechanistically, age-related accumulation of mitochondrial DNA mutations causes mitochondrial dysfunction. Treatment with mitochondria-targeted antioxidants (e.g., MitoQ) restores mitochondrial function in osteoblasts, promotes bone formation, and mitigates bone loss in aged osteoporotic mice.

In osteoclasts, disrupted mitochondrial symbiosis exhibits a “bidirectional effect”: moderate mitochondrial dysfunction promotes osteoclastogenesis, whereas severe dysfunction inhibits it. Osteoclast differentiation requires ROS as a second messenger, with mitochondria serving as the primary ROS source (Shao et al., 2024). Studies reveal that OVX mice exhibit moderately increased mitochondrial ROS production in osteoclasts, which promotes osteoclast differentiation by activating the NF-κB signaling pathway. Conversely, using mitochondrial ROS scavengers inhibits osteoclastogenesis and reduces bone loss. However, when mitochondrial function is severely impaired (e.g., by large-scale mitochondrial DNA deletions), insufficient ATP production in osteoclasts leads to suppression of their bone resorption function. This bidirectional effect suggests that mitochondrial symbiosis disruption plays a complex role in osteoporosis, necessitating tailored therapeutic strategies based on its severity (Yuan et al., 2022; Wang P. et al., 2024). Notably, this bidirectional effect of mitochondrial dysfunction in osteoclasts can be linked to specific clinical biomarkers of mitochondrial stress, which may predict the switch between pro-osteoclastogenic and anti-osteoclastogenic states. Potential predictive biomarkers include: 1) circulating levels of mitochondrial DNA (mtDNA) fragments, where moderate increases correlate with enhanced osteoclastogenesis (associated with NF-κB activation) and massive mtDNA release indicates severe mitochondrial damage and impaired bone resorption; 2) serum levels of superoxide dismutase 2 (SOD2) and peroxiredoxin 3 (PRDX3) (mitochondrial-specific antioxidants), with reduced expression reflecting moderate mitochondrial stress and pro-osteoclastogenic activity, while drastically decreased levels signal severe mitochondrial dysfunction; 3) osteoclast-specific markers such as tartrate-resistant acid phosphatase (TRAP) and cathepsin K (CTSK), where elevated levels correspond to moderate mitochondrial dysfunction (promoting osteoclastogenesis) and reduced levels align with severe mitochondrial impairment (inhibiting bone resorption). These biomarkers may provide clinical tools to stratify patients and guide targeted therapeutic interventions based on the degree of mitochondrial dysfunction.

**FIGURE 4:**
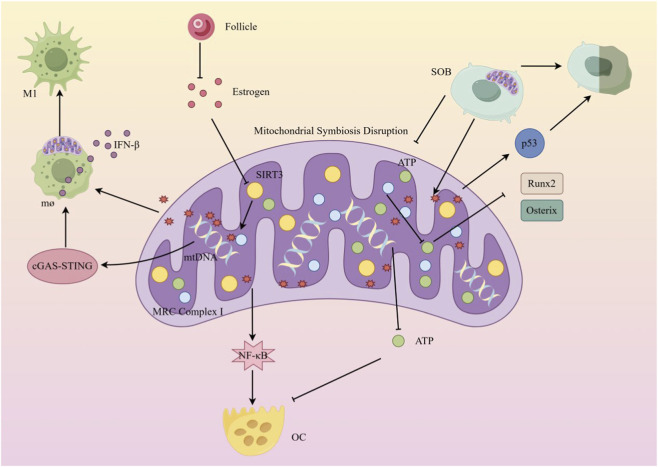
Metabolic reprogramming-bone metabolic imbalance association in mitochondrial symbiosis disruption Differential regulatory mechanisms and core signaling chains of mitochondrial symbiosis disruption in different cell types: ① At the macrophage level (top left): estrogen deficiency inhibits SIRT3 expression, leading to dysfunction of mitochondrial respiratory chain complex I (MRC Complex I), increased ROS production, and activation of the cGAS-STING pathway, promoting M1 polarization and IFN-β secretion; ② At the osteoblast level (bottom left): mitochondrial dysfunction results in insufficient ATP production, suppressing the expression of osteogenic genes such as Runx2 and Osterix, while ROS activates the p53 pathway, inducing osteoblast senescence; ③ At the osteoclast level (right): moderate mitochondrial ROS activates NF-κB to promote osteoclast differentiation, while severe dysfunction inhibits bone resorption due to ATP deficiency, reflecting a “bidirectional effect.” In the figure, “mitochondrial symbiosis disruption” serves as the central node, linking upstream triggers such as estrogen deficiency, SIRT3, and mtDNA with downstream cellular dysfunction, fully presenting the pathological network of “mitochondrial damage–metabolic reprogramming–reduced bone formation and disrupted bone resorption.”

Recent years have seen progress in developing therapeutic approaches targeting mitochondrial symbiosis disruption. For instance, the mitochondrial-targeted antioxidant MitoQ significantly restored osteoblast mitochondrial function and promoted bone formation in an OVX mouse model; while metformin, by activating the AMPK signaling pathway, improves mitochondrial metabolism in macrophages, suppresses M1 polarization, and reduces bone loss (Hsu et al., 2025; Srinivasan et al., 2010). These studies suggest that targeting mitochondrial symbiosis disruption may represent a novel therapeutic direction for osteoporosis (Figure 4).

## Treatment strategies for osteoporosis targeting the innate immune system

Given the pivotal role of the innate immune system in osteoporosis, therapeutic approaches targeting innate immune regulatory pathways have emerged as a research hotspot in this field. Currently, relevant therapeutic approaches primarily include: immunometabolic modulators, complement receptor antagonists, cytokine-targeted drugs, cell therapies, and active components from traditional Chinese medicine (Guo et al., 2021). These strategies have achieved significant progress in preclinical studies, with some already entering clinical trial phases. This section systematically summarizes research advances in these therapeutic approaches and analyzes their advantages and challenges.

## Immunometabolic modulators

Immunometabolic modulators restore normal function by regulating metabolic reprogramming in innate immune cells, thereby improving bone metabolic balance. The advantage of these drugs lies in their ability to simultaneously target multiple immune cell subsets with relatively high safety, making them an important direction for osteoporosis treatment (Ding et al., 2024).

## Glycolysis inhibitors

Glycolysis serves as the primary energy metabolism pathway for inflammatory cells such as M1 macrophages and activated neutrophils. Inhibiting glycolysis reduces proinflammatory cytokine secretion and suppresses osteoclastogenesis (Zhang S. et al., 2025). 2-Deoxyglucose (2-DG) is a classic glycolysis inhibitor that competitively blocks glucose transporters and hexokinase to inhibit glycolytic metabolism (Laussel and Leon, 2020). Preclinical Studies reveal that 2-DG significantly suppresses glycolytic activity in bone marrow macrophages from ovariectomized (OVX) mouse models, reducing TNF-α and IL-6 secretion, thereby inhibiting osteoclastogenesis and enhancing bone mineral density (BMD) (Porwal et al., 2017). Additionally, 2-DG further mitigates bone loss by inhibiting neutrophil NET formation. However, 2-DG is currently limited to preclinical research due to non-specific toxicity (neurotoxicity, hepatotoxicity) in animal models, and no clinical trials for OP have been initiated. Higher-selectivity glycolytic inhibitors (e.g., HK2 inhibitors) are under development to address toxicity issues, but their efficacy in human OP remains untested (Bao et al., 2022; DeBerardinis et al., 2007).

## Fatty acid oxidation activators

Fatty acid oxidation serves as the primary energy metabolism pathway for M2 macrophages and osteoblasts. Activating fatty acid oxidation promotes M2 macrophage polarization and enhances osteoblast function (Takano et al., 2012). Metformin, a commonly used type 2 diabetes medication, has been shown in recent studies to activate AMPK signaling pathways, thereby promoting macrophage fatty acid oxidation and inducing M2 polarization. In an ovariectomized (OVX) mouse model, metformin significantly increased the proportion of M2 macrophages in bone marrow, reduced proinflammatory cytokine secretion, and simultaneously promoted osteoblast differentiation and restored bone mass. Clinical studies reveal that long-term metformin use in type 2 diabetes patients results in significantly higher BMD and reduced fracture risk compared to non-users, suggesting metformin’s potential as a therapeutic agent for diabetes-related osteoporosis (Fan et al., 2024; Cai Y. et al., 2024). Additionally, PPARγ (Peroxisome Proliferator-Activated Receptor γ) agonists (e.g., rosiglitazone) also promote M2 macrophage polarization by activating fatty acid oxidation, demonstrating bone-protective effects in preclinical studies (Cai W. et al., 2024).

## Mitochondrial metabolic regulators

Mitochondrial metabolic regulators improve bone metabolic balance by enhancing mitochondrial function and restoring normal metabolism in innate immune cells and bone cells. MitoQ is a mitochondria-targeted antioxidant that selectively accumulates in mitochondria, scavenges mitochondrial ROS, and improves mitochondrial function (Timblin et al., 2021). Studies have demonstrated that MitoQ significantly restores mitochondrial function in osteoblasts, promotes osteoblast differentiation, inhibits osteoclastogenesis, and increases bone mineral density (BMD) in aged osteoporotic mouse models. Additionally, MitoQ reduces proinflammatory cytokine secretion by suppressing the trained immune phenotype of macrophages, further mitigating bone loss. Currently, MitoQ is being tested in clinical trials for mitochondrial diseases, and its clinical efficacy in osteoporosis requires further validation (Li et al., 2015; Srinivasan et al., 2010).

## Complement receptor antagonists

Complement receptor antagonists improve bone metabolic balance by inhibiting excessive complement system activation through blocking the binding of complement activation products (e.g., C5a) to receptors (e.g., C5aR1). These drugs offer advantages of well-defined mechanisms of action and high target specificity, making them an important research direction for osteoporosis treatment.

## C5aR1 antagonists

C5aR1 antagonists represent the most maturely studied complement-targeted drugs, demonstrating significant efficacy in multiple inflammatory disease models (Li et al., 2024). PMX53, a classic C5aR1 antagonist, competitively binds C5aR1 to inhibit C5a-mediated inflammatory responses. Preclinical Studies reveal that PMX53 significantly suppresses osteoblast apoptosis, reduces osteoclastogenesis, and increases bone mineral density (BMD) in ovariectomized (OVX) mouse models. Additionally, PMX53 mitigates bone loss by inhibiting M1 polarization of macrophages and reducing proinflammatory cytokine secretion (Cai W. et al., 2024). Avacopan, a novel oral C5aR1 antagonist, has entered early clinical trials for rheumatoid arthritis (a disease with concurrent inflammatory bone loss), showing reduced serum TNF-α/IL-6 levels and preliminary bone-protective effects—representing the only C5aR1 antagonist with clinical data relevant to bone metabolism. However, no phase III trials for primary OP have been completed, and long-term safety (e.g., infection risk) in OP patients remains unclear (Pimenta-Lopes et al., 2023). Clinical trials for avacopan in osteoporosis treatment are currently underway, positioning it as a potential first complement-targeted drug for osteoporosis therapy.

## C3aR antagonists

C3aR antagonists improve bone metabolic balance by blocking C3a-C3aR binding and inhibiting complement system activation. Studies reveal that C3aR knockout mice exhibit significantly reduced bone loss after ovariectomy (OVX), increased osteoblast numbers, and decreased osteoclast numbers. C3aR antagonists can mimic the C3aR knockout phenotype and inhibit bone loss (Li and Cui, 2025). Mechanistically, C3aR antagonists promote osteoblast differentiation by activating the Wnt/β-catenin signaling pathway while simultaneously inhibiting osteoclastogenesis (Li J. et al., 2023; Kuhn et al., 2023). Currently, C3aR antagonists remain in the preclinical research phase, and their efficacy and safety in osteoporosis require further validation.

## Cytokine-targeted drugs

Cytokine-targeted drugs improve bone metabolic balance by inhibiting inflammatory responses through blocking the function of pro-inflammatory factors such as TNF-α, IL-6, and IL-1β. These drugs have been widely used in inflammatory diseases like rheumatoid arthritis, and recent studies have revealed their potential therapeutic value in osteoporosis.

## TNF-α antagonists

TNF-α is a key pro-inflammatory factor in osteoporosis. Targeting TNF-α significantly suppresses osteoclastogenesis and reduces bone loss (Wang et al., 2023). Etanercept is a TNF-α antagonist that competitively binds to TNF-α, preventing its interaction with receptors. Studies in mouse models demonstrate that etanercept significantly reduces osteoclast numbers and increases BMD. Additionally, by inhibiting M1 polarization of macrophages, etanercept reduces pro-inflammatory cytokine secretion, further mitigating bone loss (Seki et al., 2019). Clinical studies reveal that long-term etanercept use in rheumatoid arthritis patients significantly increases BMD and reduces fracture risk, suggesting TNF-α antagonists may become therapeutic agents for inflammatory osteoporosis (Kearsley-Fleet et al., 2023). It is important to clarify that while these agents effectively target innate immune mediators to preserve bone mass in inflammatory conditions (e.g., rheumatoid arthritis-associated osteoporosis), they are not standard first-line therapies for primary postmenopausal osteoporosis (PMOP). This is primarily due to the potential increased risk of infection with long-term use and suboptimal cost-benefit ratios compared to conventional PMOP treatments, limiting their clinical utility to inflammatory bone loss rather than uncomplicated PMOP. However, prolonged use of TNF-α antagonists may increase infection risk and requires careful clinical evaluation.

## IL-6 receptor antagonists

IL-6 is another key pro-inflammatory factor in osteoporosis. Targeting the IL-6 receptor significantly inhibits osteoclastogenesis and promotes osteoblast function (Chen et al., 2025). Tocilizumab is an IL-6 receptor antagonist that competitively binds to the IL-6 receptor, thereby inhibiting IL-6-mediated signaling pathways (Silberstein, 2021). Studies have shown that in mouse models of ischemic bone necrosis, tocilizumab significantly activates the STAT3 signaling pathway in osteoblasts, promotes osteoblast differentiation, inhibits osteoclast formation, and increases BMD (Kuroyanagi et al., 2023). Clinical studies in polymyalgia rheumatica (PMR) patients treated with tocilizumab demonstrated significant increases in serum bone formation markers (e.g., PINP) and decreases in bone resorption markers (e.g., CTX), suggesting IL-6 receptor antagonists may serve as therapeutic agents for osteoporosis (Carvajal Alegria et al., 2021). Similar to TNF-α antagonists, tocilizumab and other IL-6 receptor antagonists are primarily indicated for inflammatory bone loss conditions rather than uncomplicated primary postmenopausal osteoporosis. Their use in PMOP is not recommended as first-line therapy due to concerns about infection risks and cost-effectiveness, with their clinical value centered on targeting innate immune-driven inflammatory bone metabolism disorders. Clinical trials evaluating tocilizumab for osteoporosis treatment are currently underway, with its efficacy and safety requiring further validation.

## IL-1β antagonists

IL-1β is a potent pro-inflammatory factor that promotes osteoclastogenesis by activating the NF-κB signaling pathway (Yao et al., 2021). Anakinra, an IL-1β antagonist, inhibits IL-1β-mediated inflammatory responses by competitively binding to the IL-1 receptor (Sullender et al., 2024). Studies have demonstrated that anakinra significantly suppresses osteoclastogenesis and increases BMD in an OVX mouse model. Additionally, anakinra reduces IL-1β secretion by inhibiting NLRP3 inflammasome activation, thereby further mitigating bone loss (Wang et al., 2025). Clinical studies in patients with the rare disease familial Mediterranean fever showed significant BMD increases after long-term anakinra treatment, suggesting IL-1β antagonists may become therapeutic agents for osteoporosis. However, their clinical application is limited by short-term efficacy and high costs, necessitating further optimization of treatment regimens (Ocak et al., 2025; Chen et al., 2003).

## Cell therapy

Cell therapy restores immune balance in the bone microenvironment by infusing functional innate immune cells or their precursor cells, thereby improving bone metabolism. This approach offers advantages of high targeting specificity and sustained efficacy, representing an emerging direction in osteoporosis treatment (He et al., 2025).

## M2 macrophage infusion

M2 macrophages promote bone repair and regeneration by secreting anti-inflammatory and pro-osteogenic factors (Chen et al., 2022b). Studies show that infusing in vitro-induced M2 macrophages into ovariectomized (OVX) mice significantly increases the proportion of M2 macrophages in bone marrow, reduces pro-inflammatory factor secretion, promotes osteoblast differentiation, and enhances bone mineral density (BMD) (Zhang et al., 2024). Mechanistically, TGF-β secreted by M2 macrophages activates the Smad signaling pathway in osteoblasts, promoting their differentiation. Meanwhile, BMP-2 secreted by M2 macrophages directly promotes the differentiation of mesenchymal stem cells (MSCs) into osteoblasts (Zhang et al., 2017). Currently, M2 macrophage infusion remains in the preclinical research phase, with its efficacy and safety in osteoporosis requiring further validation.

## Infusion of regulatory neutrophils

Regulatory neutrophils constitute an anti-inflammatory neutrophil subset that maintains immune homeostasis by suppressing NET formation and proinflammatory cytokine secretion (Doz-Deblauwe et al., 2024). Studies have shown that infusion of regulatory neutrophils into rats significantly reduces NET levels in bone marrow, inhibits osteoclastogenesis, promotes osteoblast function, and increases bone mineral density (BMD) (Isojima et al., 2025). Mechanistically, IL-10 secreted by regulatory neutrophils promotes osteoblast differentiation by activating the STAT3 signaling pathway in osteoblasts; while antioxidant enzymes released by regulatory neutrophils (e.g., superoxide dismutase) scavenge ROS, protecting osteoblasts from oxidative damage (Guo H. et al., 2025). Currently, regulatory neutrophil infusion remains in the preclinical research phase, with promising prospects for its application in osteoporosis.

## Active components of Traditional Chinese Medicine

Active components of traditional Chinese medicine exhibit multi-targeted and multi-pathway effects with high safety profiles, offering unique advantages in osteoporosis treatment. Recent studies reveal that multiple active components can regulate innate immune cell function and improve bone metabolic balance, making them important resources for osteoporosis therapy development.

## Total flavonoids of dipsacus asper

Total flavonoids of Dipsacus asper are the primary bioactive components of the herb, consisting of monomers including naringin and neohesperidin. Their pharmacological effects relevant to bone health are mediated through specific molecular targets rather than traditional anecdotal uses. In bone immune regulation, these flavonoids balance the polarization of bone marrow macrophages. In ovariectomized (OVX) or high-glucose bone loss models, they upregulate PPARδ expression in macrophages to promote mitochondrial fatty acid oxidation, inhibit the glycolysis-dependent NF-κB pathway, and induce M2 polarization (Xie et al., 2021). IL-10 and TGF-β secreted by M2 cells inhibit osteoclast precursor differentiation, reduce TRAP-positive osteoclasts, and decrease MMP9 and CTSK expression. Concurrently, these flavonoids downregulate TLR4/MyD88 pathway activity, reduce LPS-induced TNF-α and IL-6 release, and disrupt the “inflammation-osteoclast” cycle (Zhu et al., 2012; Chen et al., 2021).

At the osteogenic regulatory level, DFTF promotes bone differentiation by activating the BMP/Smad signaling pathway. The core monomer naringin binds to osteoblast BMPRIA, enhancing receptor dimerization and Smad1/5/8 phosphorylation. Phosphorylated Smad1/5/8 forms a complex with Smad4 and translocates to the nucleus, promoting Runx2, Osx, and Col1a1 transcription. This accelerates osteoblast transformation and increases bone matrix synthesis (Dong et al., 2020; Sun et al., 2021). Furthermore, in fracture healing, total flavonoids from Psoralea corylifolia promote callus formation and angiogenesis: accelerating chondrocyte differentiation into osteoblasts to enhance endochondral ossification; upregulating VEGF expression to induce periosteal vascularization around the callus, thereby shortening healing time (Huang et al., 2018). Currently, Phase II clinical trials of total Salvia miltiorrhiza flavonoids for primary osteoporosis and delayed fracture healing have preliminarily confirmed their safety and demonstrated efficacy in improving bone density. However, their long-term therapeutic effects require further validation through Phase III clinical trials.

## Icariin

Icariin is the primary bioactive component of Epimedium, with well-defined molecular mechanisms underlying its bone-protective effects. In diabetic osteoporosis (DOP) mouse models, icariin significantly promotes the polarization of bone marrow M2 macrophages, reduces pro-inflammatory factor secretion, and simultaneously activates the Wnt/β-catenin signaling pathway in osteoblasts, thereby enhancing osteoblast differentiation and increasing bone mineral density (BMD) (Chai et al., 2022). Mechanistically, icariin upregulates PPARγ expression in macrophages to promote fatty acid oxidation and M2 polarization; concurrently, it inhibits GSK-3β activity in osteoblasts to reduce β-catenin degradation, activating the Wnt/β-catenin signaling pathway (Wang et al., 2019; Wang H. et al., 2024). Clinical trials investigating icariin for OP treatment are currently underway, with its efficacy and safety requiring further validation.

## Artificial tiger bone powder

Artificial tiger bone powder is a scientifically formulated substitute for natural tiger bone, with its bone-protective effects attributed to its specific composition collagen protein, calcium carbonate, calcium phosphate, and essential amino acids (e.g., proline, glycine). These components directly contribute to its molecular mechanisms of action in bone metabolism. In OVX-induced osteoporosis mouse models and hormone-induced osteoporosis rat models, artificial tiger bone powder significantly suppresses excessive osteoclast activation and reduces bone resorption, while promoting osteoblast proliferation and function, enhancing bone mineral density (BMD) and trabecular number (Tb.N), and improving bone microstructural integrity (Shen et al., 2022).

Mechanistically, artificial tiger bone powder upregulates bone morphogenetic protein 2 (BMP-2) expression in osteoblasts to activate the BMP/Smad signaling pathway, thereby promoting transcription of osteogenesis-related genes (Runx2, Osx, Col1a1) and accelerating bone matrix synthesis and mineralization. Simultaneously, it inhibits the expression of receptor activator of nuclear factor kappa-B ligand (RANKL) in osteoclasts, reduces the RANKL/osteoprotegerin (OPG) ratio, and decreases the differentiation of osteoclast precursors into mature osteoclasts, thereby suppressing bone resorption (Ren et al., 2021). Furthermore, its calcium and phosphorus components regulate serum calcium-phosphorus metabolism balance, enhancing osteoblast calcium uptake and utilization to reinforce bone-forming effects (Shu and Zhang, 2022). Currently, Phase II clinical trials of artificial tiger bone powder for treating primary osteoporosis and osteoarthritis have yielded preliminary results, showing effective alleviation of bone pain symptoms and increased lumbar spine and hip BMD without significant adverse reactions. However, its long-term efficacy stability and intervention effects on severe osteoporosis patients require further validation through Phase III clinical trials.

## Future prospects and challenges

Although osteoporosis therapies targeting innate immunity have advanced, core challenges remain: First, insufficient target specificity—many drugs (e.g., TNF-α antagonists, C5aR1 antagonists) may impair normal immunity and bone metabolism (e.g., TNF-α inhibition delays fracture healing, while C5aR1 antagonists induce cardiovascular side effects), necessitating the development of bone tissue- or cell-specific drugs. Second, treatment response heterogeneity arises from patient genetic backgrounds, age, and comorbidities (e.g., diabetes), requiring biomarker-guided precision medicine. Third, long-term safety remains unvalidated. Most drugs are in preclinical/early stages, complement antagonists increase infection risk, and cell therapies face complex preparation and high costs, necessitating process optimization.

Future breakthroughs may emerge from four directions: First, leveraging nanotechnology, CRISPR editing (e.g., CXCR4 modification), and single-cell sequencing to develop bone-targeted drugs and identify specific targets; Second, exploring combination therapies (e.g., complement antagonists + RANKL inhibitors, immunometabolic modulators + traditional Chinese medicine) to synergistically improve bone metabolism through multi-target approaches while reducing dosages and side effects; Third, decipher emerging mechanisms like trained immunity (epigenetic drugs) and mitochondrial symbiosis disruption (metabolic enzyme drugs), leveraging single-cell multi-omics to uncover novel pathways; Fourth, conduct large-scale clinical trials incorporating diverse patient subtypes, stratified by biomarkers, with long-term follow-up to validate efficacy and safety.

In summary, innate immunity (cells + complement) regulates bone metabolic balance through multiple mechanisms, representing a key pathway in osteoporosis pathogenesis. Existing targeted strategies show promise but require overcoming the aforementioned challenges. Advancing translational research through specific drugs, combination therapies, mechanism elucidation, and clinical trials will ultimately deliver more effective and safer treatment options for patients, providing robust support for global osteoporosis prevention and management.

## Conclusion

In conclusion, this review systematically explores the “innate immunity-bone metabolism” regulatory network, focusing on innate immune cell dysfunction, complement overactivation (C5a/C5aR1 axis), trained immunity, and mitochondrial symbiosis disruption as key drivers of bone metabolic imbalance. The summarized therapeutic strategies, including immunometabolic modulators, complement antagonists, and TCM bioactive components, show promise but require further clinical validation. Future research should prioritize overcoming target specificity issues and translating preclinical findings to clinical practice, ultimately providing more effective and safe treatments for OP.
